# The Ycx1 protein encoded by the yeast YDL206W gene plays a role in calcium and calcineurin signaling

**DOI:** 10.1016/j.jbc.2023.104647

**Published:** 2023-03-24

**Authors:** Grace M. Lee, Fangli Weng, Juliana Cranley, Abhinav Rajasekhar, Matthew Stoeckel, Thomas Kane, Renata Tisi, Yuqi Wang

**Affiliations:** 1 Department of Biology, Saint Louis University, St Louis, Missouri, USA; 2 Department of Biotechnology and Bioscience, University of Milano-Bicocca, Milan, Italy

**Keywords:** YDL206W, Ycx1, calcineurin, cell wall, calcium, yeast, pheromone signaling

## Abstract

Calcium is ubiquitously present in all living cells and plays important regulatory roles in a wide variety of biological processes. In yeast, many effects of calcium are mediated *via* the action of calcineurin, a calcium/calmodulin-dependent protein phosphatase. Proper signaling of calcium and calcineurin is important in yeast, and the calcineurin pathway has emerged as a valuable target for developing novel antifungal drugs. Here, we report a role of YDL206W in calcium and calcineurin signaling in yeast. YDL206W is an uncharacterized gene in yeast, encoding a protein with two sodium/calcium exchange domains. Disrupting the YDL206W gene leads to a diminished level of calcium-induced activation of calcineurin and a reduced accumulation of cytosolic calcium. Consistent with a role of calcineurin in regulating pheromone and cell wall integrity signaling, the *ydl206wΔ* mutants display an enhanced growth arrest induced by pheromone treatment and poor growth at elevated temperature. Subcellular localization studies indicate that YDL206W is localized in endoplasmic reticulum and Golgi. Together, our results reveal YDL206W as a new regulator for calcineurin signaling in yeast and suggest a role of the endoplasmic reticulum and Golgi in regulating cytosolic calcium in yeast.

The budding yeast, *Saccharomyces cerevisiae*, is one of the most widely used model organisms in biology. Because of the remarkable efficiency of homologous recombination in yeast and the fact that it can exist as stable haploids, resources have been developed to allow the investigation of every yeast gene in a systematic way. These include the systematic deletion library for nonessential genes (1), the conditional disruption library for essential genes (2), and the epitope-tagged yeast ORF libraries (3, 4). Successful applications of these powerful resources over the years have revealed remarkable insights on all aspects of biology and the function of many previously uncharacterized genes.

Despite tremendous efforts by yeast researchers over the years, there still exists a large number of genes in yeast with unknown function. One such gene is YDL206W, which is listed as an uncharacterized ORF encoding a putative protein of unknown function. Based on Pfam domain analysis, YDL206W contains domains belonging to the central ion-binding domain superfamily found in the sodium/calcium exchangers (NCX) (Fig. 1), suggesting a potential role of this protein in regulating calcium signaling in yeast. The goal of this study is to test this hypothesis and investigate if YDL206W is indeed involved in regulating calcium signaling.

**Figure 1:**
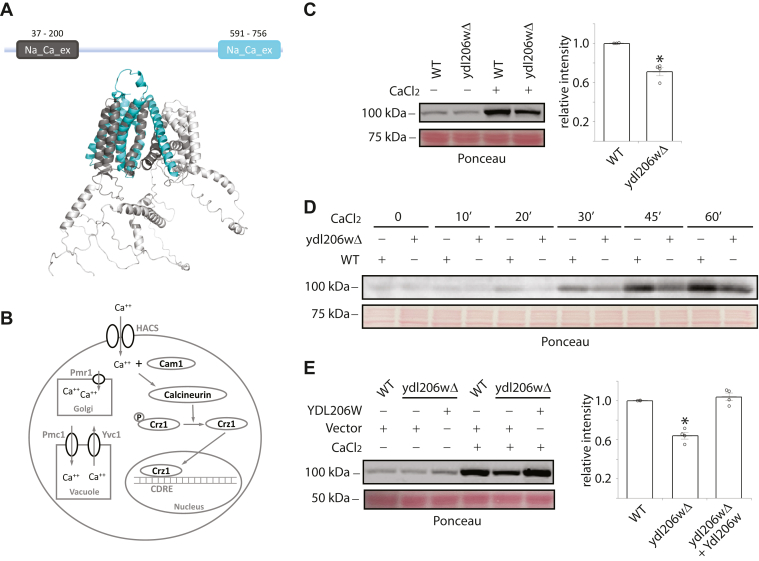
**Disrupting YDL206W diminishes calcineurin-dependent gene expression.***A,* Pfam analysis and the AlphaFold-predicted structure model of YDL206W. *B,* diagram of calcium–calcineurin pathway in yeast. Calcium entry *via* high-affinity Ca^2+^ influx system (HACS) in the plasma membrane and/or release from vacuole *via* Yvc1 leads to an increase in cytosolic calcium, which binds the calcium sensor calmodulin (Cam1). Ca^++^–calmodulin in turn activates the phosphatase calcineurin, which dephosphorylates Crz1, a transcription factor that binds to calcium–calcineurin-dependent regulatory element (CDRE) and promotes gene expression. *C,* wildtype or *ydl206wΔ* cells were transformed with a plasmid that expresses a reporter gene LacZ under the control of four tandem CDRE (CDRE-LacZ). Cells were grown to midlog phase and treated or not treated with 80 mM CaCl_2_ for 1 h, and whole cell extracts were prepared and separated on 8% SDS-PAGE, transferred to a nitrocellulose membrane, and immunoblotted with anti–beta-galactosidase antibody (catalog no.: Z-3781; Promega). Equal loadings were conformed with Ponceau S staining. The relative level of beta galactosidase in CaCl_2_-treated cells was quantified using ImageJ with the level of WT set as 1. Quantified results from three independent experiments are shown on the *right*. *D,* the same cells as in *C* were grown to midlog phase and treated with 80 mM CaCl_2_ for the indicated time. Whole cell extracts were analyzed by Western blotting using anti–beta-galactosidase antibodies. *E,* wildtype or *ydl206wΔ* cells were transformed with CDRE-LacZ reporter and either an empty vector or a plasmid that expresses YDL206W under its own promoter. Midlog phase cells were processed as described previously in *C*, and the quantified results from CaCl_2_-treated cells from three independent experiments are shown on the *right*.

Ca^2+^ is a key factor that regulates many biological processes in all eukaryotes especially for the processes that require a rapid onset and/or adaptation to environmental stimulus (5). In yeast, proper calcium signaling is required for growth, morphogenesis, cell wall integrity, responses to mating pheromone, and adaptation to hyperosmotic stress (6, 7, 8). In normal yeast cells, the basal cytosolic concentration of calcium is maintained at a very low level (around 50–200 nM) (9). In response to internal or external stimulus, cytosolic calcium level can rise very rapidly *via* influx from plasma membrane and/or release from the intracellular storage sites (6). In yeast, the major calcium storage site is the vacuole, a lysosome-like organelle, which stores about 90% of intracellular calcium, whereas the endoplasmic reticulum (ER) and Golgi also maintain a relatively high level of calcium (6, 10). Increased cytosolic calcium is sensed by calmodulin, a protein with the calcium-binding EF-hand motifs (11, 12). In its calcium-bound form, calmodulin binds and activates a variety of downstream proteins that include calcineurin, a highly conserved protein phosphatase (13, 14) (Fig. 1). Activated calcineurin in turn dephosphorylates Crz1 (15, 16), a transcription factor, leading to its rapid translocation from the cytosol to the nucleus to promote the expression of genes that include ion pumps and enzymes for cell wall synthesis (17). The rise of cytosolic calcium is transient, and the concentration of calcium goes back to its basal level shortly thereafter (6).

The proper cytosolic level of calcium is maintained by channels, transporters, and pumps. In yeast, influx of calcium through the plasma membrane is mainly mediated by the high-affinity calcium transporter, a protein complex composed of Mid1 (18), Cch1 (18), and Ecm7 (19), and the release of calcium from its major storage site vacuole is achieved *via* Yvc1, a vacuolar cation channel (20, 21). Conversely, sequestering the excess calcium into storage is mediated by Pmr1, a P-type ATPase required for Ca^2+^ transport into the Golgi (10, 22), and Vcx1, a vacuolar Ca^2+^/H^+^ exchanger that transports Ca^2+^ into the vacuole (23).

In mammalian cells, especially in cardiac myocytes, one major player in controlling cytosolic calcium is the NCX, which uses the energy from the sodium gradient to move calcium (5). In normal cells, the sodium concentration is higher in the extracellular space. NCX imports sodium down its gradient to the cell and exports calcium concurrently (24). In this so-called forward mode, NCX serves to expel excess calcium out of the cytosol. Under certain conditions such as an abnormally higher intracellular buildup of sodium, NCX acts in the reverse mode by bringing calcium into the cells instead (25). Notably, a NCX protein has not been identified in yeast. The yeast YDL206W encodes a putative protein with structural motifs present in NCX proteins, which prompted us to investigate its potential role in regulating intracellular calcium levels and calcium signaling. Using the well-established probes, we find that deleting YDL206W gene leads to a diminished expression of calcineurin-regulated genes, a reduced level of cytosolic calcium, and an altered adaptation to pheromone and cell wall stress. We also find that YDL206W is localized in ER and Golgi and is required for maintaining the proper level of chitin on the cell surface. Our findings thus reveal a function of YDL206W, a previously uncharacterized protein, in the calcium–calcineurin pathway.

## Results

### Disrupting YDL206W diminishes calcineurin-dependent gene expression

YDL206W is an uncharacterized ORF in the yeast genome. Pfam analysis of its protein sequence indicates the presence of two sodium/calcium exchanger (NCX) domains, suggesting a potential role of this protein in regulating calcium signaling (Fig. 1*A*). Calcineurin is a calcium–calmodulin-activated phosphatase that dephosphorylates and activates Crz1, a transcription factor that promotes the expression of genes containing the calcineurin-dependent response element (CDRE) (16) (Fig. 1*B*). To examine if YDL206W is involved in calcium signaling, we first used a reporter gene (LacZ) under the control of four tandem repeats of the CDRE (4XCDRE-LacZ) (16, 26) and compared the behavior of this reporter in wildtype *versus* the *ydl206wΔ* mutants, with or without the addition of 80 mM of CaCl_2_ in the media. As shown in Figure 1*C*, in both the wildtype and the *ydl206wΔ* mutant, the addition of CaCl_2_ caused an increase in the level of beta-galactosidase, the product of LacZ gene. However, the level of increase is smaller in the *ydl206wΔ* mutant, which is consistent with a role of YDL206W in potentiating calcium–calcineurin signaling. Time-course experiments indicated that the *ydl206wΔ* mutant has a lower 4XCDRE activity than wildtype in all time points examined (Fig. 1*D*). To ensure the effect we saw in the *ydl206wΔ* mutant is caused by a loss of YDL206W, we cloned the YDL206W gene into a single-copy yeast expression plasmid and transformed the plasmid into the *ydl206wΔ* mutant. As shown in Figure 1*E*, expressing YDL206W from the plasmid was able to restore CaCl_2_-induced increase of the 4XCDRE activity in the *ydl206wΔ* mutant to the same level as in wildtype, indicating the effect displayed in the *ydl206wΔ* mutant is indeed because of the loss of YDL206W.

### A role of YDL206W in regulating cytosolic level of calcium

**Figure 2:**
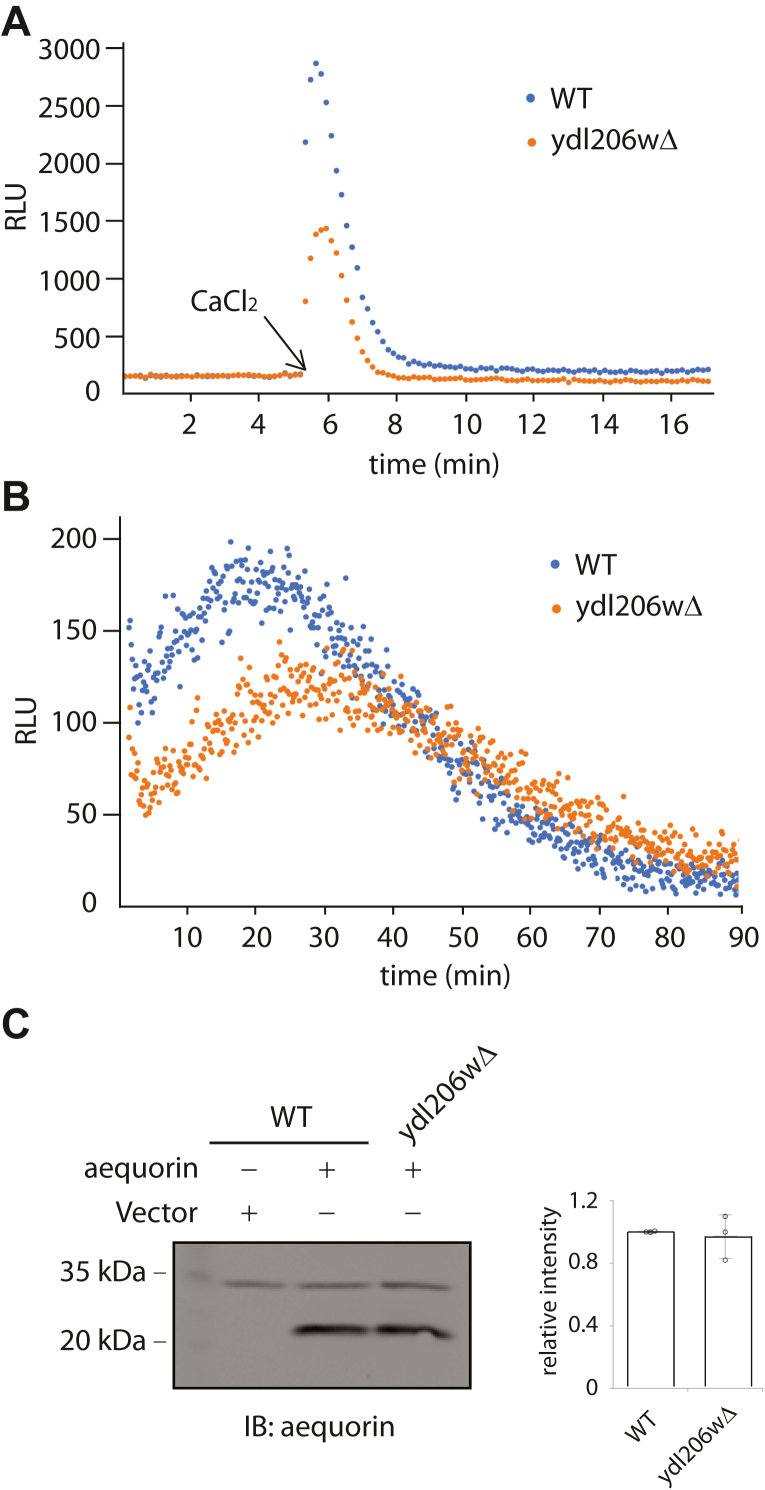
**Intracellular calcium level.***A,* wildtype or *ydl206wΔ* cells transformed with a plasmid expressing apoaequorin were grown to midlog phase. Equal amounts of cells were incubated with coelenterazine for 30 min. After a stable baseline measurement of bioluminescence was achieved, cells were treated with 80 mM CaCl_2,_ and the resulting bioluminescence was recorded every 14 s. *B,* the same cells as described in *A* were grown to midlog phase, treated with 3 μM α-factor for 15 min and incubated with coelenterazine for 30 min, and the bioluminescence was measured every 14 s. *C,* the same cells as described previously were cultured to midlog phase. Whole cell extracts were separated on 12% SDS-PAGE and immunoblotted with antiaequorin to examine the level of aequorin expression. Quantified results from four independent experiments are shown on the *right*.

The effect of disrupting YDL206W gene on calcineurin-dependent gene expression is consistent with a role of YDL206W in the control of cytosolic level of calcium. To test this, we used a well-established apoaequorin-based bioluminescence assay and compared the cytosolic calcium levels in the wildtype and the *ydl206wΔ* mutants. In this assay, cells were transformed with a plasmid that expresses apoaequorin. Transformed cells were grown to midlog phase and then incubated with coelenterazine for a short period to produce functional aequorins, which generate a bioluminescence signal upon binding to cytosolic calcium ions (27). It has been shown in the literature that either increasing extracellular calcium or treating cells with mating pheromone alpha factor can lead to a transient increase in the cytosolic calcium level (27, 28). Thus, we decided to examine if YDL206W is involved in either extracellular calcium- or pheromone-induced increase in the cytosolic calcium. As shown in Figures 2*A* and S1*A*, an addition of 80 mM CaCl_2_ into the media indeed results in a rapid and transient increase in the bioluminescence signal, which corresponds to the rapid and transient increase in the cytosolic calcium level as expected. Interestingly, the level of bioluminescence signal is clearly lower in the *ydl206wΔ* cells, indicating that a complete increase in cytosolic calcium requires YDL206W. Similarly, the pheromone-induced rise in the level of cytosolic calcium is also partially dependent on YDL206W (Figs. 2*B* and S1*B*).

### Physiological function of YDL206W

**Figure 3:**
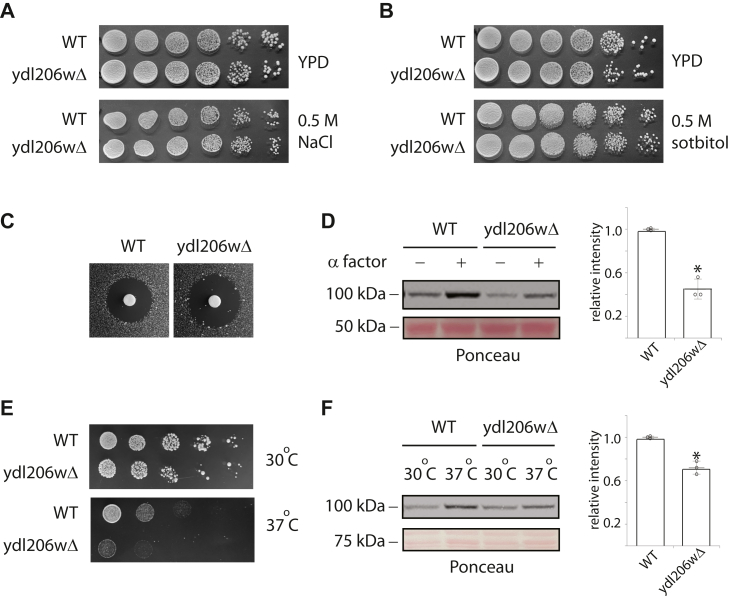
**Examining physiological functions of YDL206W.***A,* wildtype or *ydl206wΔ* cells were grown in YPD, serially diluted, plated to either YPD plate (*top panel*) or YPD plate with 0.5 M NaCl (*lower panel*), grown for 2 days at 30 °C, and imaged. *B,* the same experiments were done as in *A*, except that 0.5 M sorbitol was used as a stressor. *C,* wildtype or *ydl206wΔ* cells transformed with CDRE-LacZ reporter were plated to SCD-uracil plate, and a paper disc with 15 μl of 3 mM α-factor was placed in the nascent lawn of cells. The plates were imaged after 2 days of incubation at 30 °C. The G1 arrest induced by α-factor led to a zone of inhibition around the disc, and the size of the growth inhibition indicates the sensitivity to α-factor. *D,* midlog phase cells were treated or not treated with 3 μM α-factor for 1 h. Whole cell extracts were immunoblotted with anti–beta-galactosidase antibody. Quantified results from three independent experiments are shown on the *right*. *E,* cells were serially diluted, plated, grown at either 30 °C or 37 °C for 2 days, and imaged. *F,* cells transformed with CDRE-LacZ were grown to midlog phase and shifted to 37 °C for 3 h. Whole cell extracts were immunoblotted with anti–beta-galactosidase antibody. Quantified results from three independent experiments are shown on the *right*. CDRE, calcineurin-dependent response element; YPD, yeast extract–peptone–dextrose.

Next, we sought to identify any physiological roles YDL206W might have. In particular, we wanted to investigate if YDL206W plays any role in regulating signaling pathways that involve calcium. To this end, we compared the behaviors of wildtype *versus* the *ydl206wΔ* mutants in their responses to hyperosmotic stress, mating pheromone, and cell wall stress, which are all processes that have been documented or suggested to involve calcium and calcineurin (14, 20, 29, 30, 31). First, we compared the responses to hyperosmotic stress. For this purpose, we compared the sensitivity of wildtype cells and the *ydl206wΔ* mutants to 0.5 M NaCl. As shown in Figure 3*A*, no difference between the wildtype and the mutants was observed. We also compared the sensitivity of wildtype cells and the *ydl206wΔ* mutants to other osmolytes including 0.5 M KCl and 0.5 M sorbitol. Again, no clear differences between the wildtype and the mutants were observed (Fig. S1). Thus, it appears that YDL206W is not required for cellular tolerance to hyperosmotic stress. Next, we examined responses to mating pheromone. As shown in Figure 3*C*, the *ydl206wΔ* mutants were more sensitive to pheromone, as indicated by a larger growth inhibition zone induced by pheromone α-factor. Consistent with a previous report (29), pheromone treatment led to an increase in the activity of calcineurin as measured by 4XCDRE-LacZ reporter (Fig. 3*D*). Notably, the level of increase was clearly less in the *ydl206wΔ* mutants. Finally, we compared the responses of wildtype and the *ydl206wΔ* mutants to cell wall stress. To this end, we examined the effects of raising the temperature from 30 to 37 °C, which is one of the most commonly used and physiologically relevant cell wall stresses. As shown in Figure 3*E*, raising the temperature led to an increase in the activity of calcineurin, as measured by 4XCDRE-LacZ reporter (32), and once again, the level of increase was smaller in the *ydl206wΔ* mutants. Consistent with the notion that proper calcineurin signaling is important for cell wall maintenance and cell survival at elevated temperature (33), the *ydl206wΔ* mutants showed a clear growth defect compared with wildtype cells at 37 °C (Fig. 3*F*).

### Subcellular localization of YDL206W

To gain a better understanding how YDL206W controls cytosolic calcium level and calcineurin activity, we sought to examine its subcellular localization. For this purpose, we chose to insert a GFP tag to YDL206W and monitor the localization of GFP-tagged YDL206W *via* confocal microscopy. Initially, we thought to use the AlphaFold predicted structure model of YDL206W to guide our tagging strategy. The structural model (Fig. 1) predicated the existence of an internal unstructured loop region between two alpha helices in YDL206W. We reasoned that inserting GFP there may have minimal effects on impacting the normal localization of the protein. Unfortunately, the fusion protein with GFP inserted in the loop region between residues 232 and 233 of YDL206W was not expressed well enough for detection, either *via* Western blotting or confocal microscopy, suggesting inserting an internal GFP tag might negatively impact the folding and/or stability of the protein.

**Figure 4:**
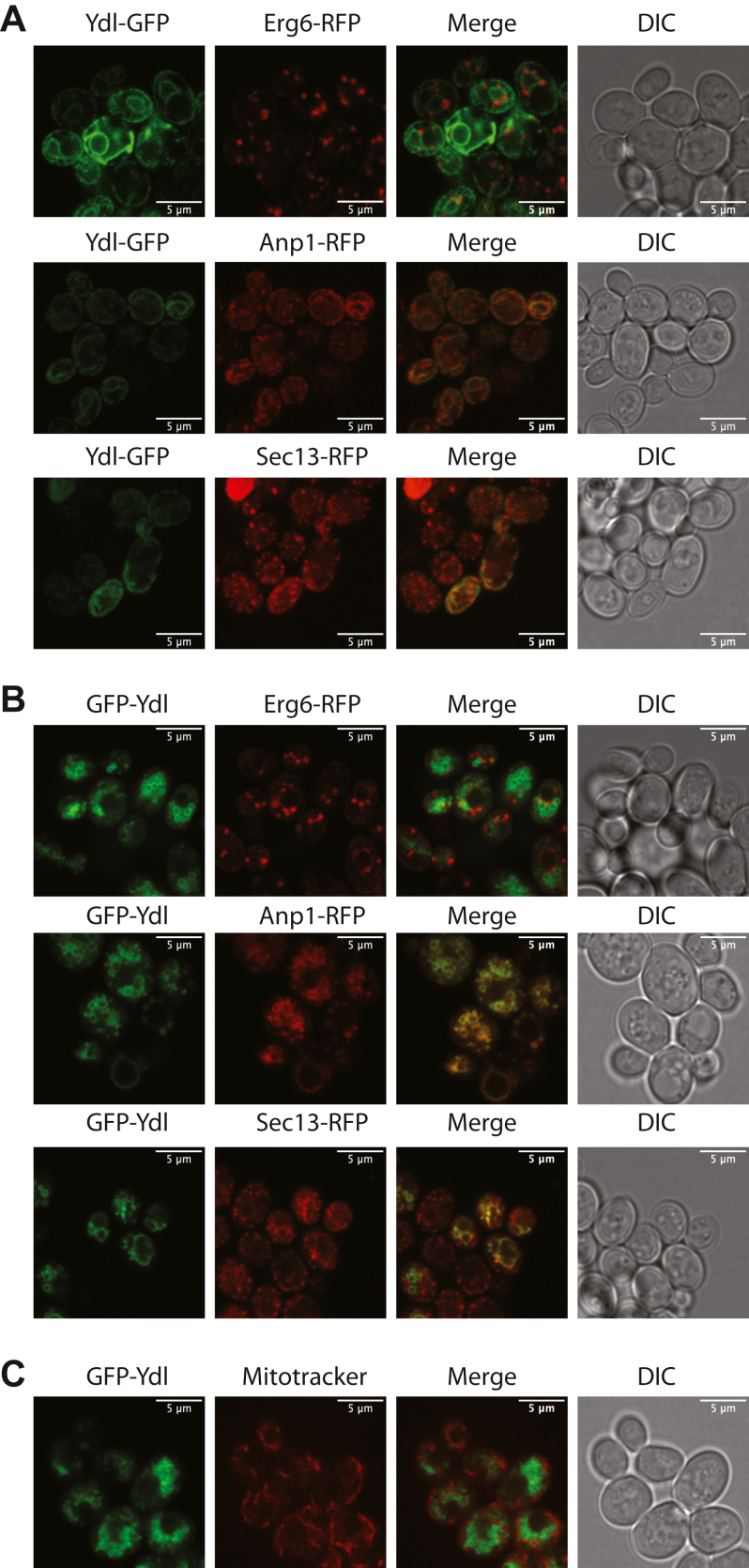
**Subcellular localization of YDL206W.***A,* wildtype cells with chromosomally tagged Erg6-RFP, Anp1-RFP, or Sec13-RFP were transformed with plasmids expressing C-terminally GFP-tagged YDL206W (YDL206W-GFP) and grown to midlog phase. The cells were subjected to confocal microscopy analysis to reveal the signals from GFP (*green*) and RFP (*red*), respectively. Overlay of GFP and RFP signals was conducted to examine possible colocalization. *C,* wildtype cells with chromosomally tagged Erg6-RFP, Anp1-RFP, or Sec13-RFP were transformed with plasmids expressing N-terminally GFP-tagged YDL206W (GFP-YDL206W) and grown to midlog phase. The cells were subjected to confocal microscopy analysis to reveal the signals from GFP (*green*) and RFP (*red*), respectively. Overlay of GFP and RFP signals was conducted to examine possible colocalization. *C,* wildtype cells transformed with N-terminally GFP-tagged YDL206W were grown to midlog phase and incubated with MitoTracker Red CM-H2X dye for 15 min, and fluorescence images were taken on a confocal microscope. RFP, red fluorescent protein.

We then decided to add GFP tag to either the C terminus or the N terminus of the protein. As shown in Figure 4*A*, the C-terminally tagged YDL206W (YDL206W-GFP) shows distinct localization inside the cells. To discern the identity of the localization, we conducted colocalization studies using three markers: Erg6-red fluorescent protein (RFP) (lipid particle), Anp1-RFP (Golgi apparatus), and Sec13-RFP (ER to Golgi). As shown in Figure 4*A*, there was substantial colocalization between YDL206W-GFP and Anp1-RFP as well as between YDL206W-GFP and Sec13-RFP, indicating that a substantial amount of the C-terminally tagged YDL206W is present in both ER and Golgi. We then analyzed the localization of the N-terminally tagged YDL206W (GFP-YDL206W), which is also present inside the cells (Fig. 4*B*). Similar to C-terminally tagged YDL206W (YDL206W-GFP), GFP-YDL206W showed substantial colocalization with both Anp1-RFP and Sec13-RFP (Fig. 4*B*). While both GFP-YDL206W and YDL206W-GFP showed colocalizations with both Anp1-RFP and Sec13-RFP, the exact localizations of GFP-YDL206W and YDL206W-GFP did not appear to be identical.

Discrepancies in localizations between the N-terminally GFP-tagged and the C-terminally GFP-tagged proteins have been previously reported with Vcx1 and Vnx1 as good examples (34, 35). In both cases, the N-terminally GFP-tagged proteins are localized in the vacuolar membrane, whereas the C-terminally GFP-tagged proteins are found in the ER (34, 35). It appears that tagging GFP to the C terminus of a membrane protein can often lead to ER retention and thus mislocalization of the protein (36). Based on this precedent, it is likely that the localization of GFP-YDL206W reflects more closely to that of the endogenous untagged protein. In mammals, NCXs can be found in mitochondria and the nucleus. To investigate if YDL206W may also be present in mitochondria, we stained cells expressing GFP-YDL206W with MitoTracker Red CM-H2X, a dye specific for the functional mitochondria. As shown in Figure 4*C*, we were not able to detect colocalization between GFP-YDL206W and MitoTracker Red. Taken together, we suggest that YDL206W is localized to the ER and Golgi.

### A role of YDL206W in regulating chitin level

**Figure 5:**
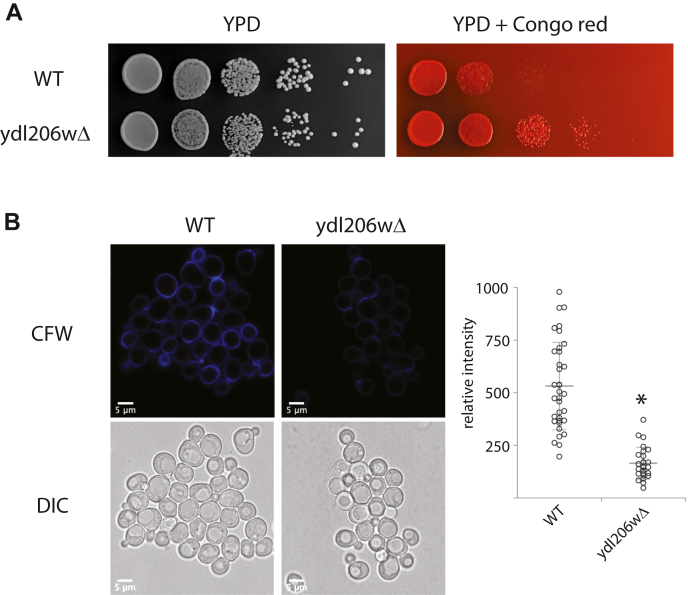
**YDL206W regulates chitin level.***A,* wildtype or *ydl206wΔ* cells were serially diluted, plated to either YPD or YPD plus 50 mg/ml Congo *red*, grew at 30 °C for 2 days, and imaged. *B,* cells were grown to midlog phase, stained with Calcofluor *white*, and imaged using confocal microscope. The same microscope settings were used for capturing the images to ensure consistency. The relative level of the fluorescence signal was quantified using ImageJ by circling each cell and measuring the integrated intensity of the cell. YPD, yeast extract–peptone–dextrose.

Previous high-throughput studies reveal that YDL206W genetically interacts with *CHS5* (37, 38), which encodes for a component of the exomer complex that is involved in the export of selected proteins including chitin synthase Chs3, from the Golgi to the plasma membrane (39). Given that YDL206W is localized in Golgi and regulates calcium signaling, we reasoned that it may affect the function of Chs5 and thus the trafficking of Chs3 from Golgi to plasma membrane, which may result in a diminished level of chitin on the cell surface. To test this, we first examined the sensitivity of *ydl206wΔ* mutant to a chitin-binding dye Congo red. A mutant with a lower level of chitin would be expected to show resistance to the harmful effect of the dye. As shown in Figure 5*A*, the *ydl206wΔ* mutant indeed is more resistant to Congo red. To monitor the chitin content more directly, we stained the cells with Calcofluor white, another chitin-binding dye that is fluorescent, thus permitting us to directly visualize the binding in live cells. As shown in Figure 5*B*, we were able to detect Calcofluor white binding in both wildtype and the *ydl206wΔ* mutants, but the level of Calcofluor white binding in the *ydl206wΔ* mutants is substantially lower.

## Discussion

In this work, we have characterized YDL206W, an ORF in yeast with unknown function. Several lines of evidence indicate that YDL206W plays a role in controlling calcium and calcineurin signaling in yeast. Using well-established calcineurin-dependent gene transcription reporter assays, we find that the *ydl206wΔ* mutants have a lower level of calcineurin activity, a defect that can be rescued by plasmid-borne YDL206W gene. Measurement of cytosolic calcium levels revealed that the magnitude of calcium spikes induced by both exogenous calcium and pheromone treatment is lower in the *ydl206wΔ* mutants. Physiologically, the *ydl206wΔ* mutant displays phenotypes that are consistent with reduced calcium–calcineurin signaling, which include a decreased adaptation to both mating pheromone and thermal stress. Notably, YDL206W appears to be localized in ER and Golgi and is required for accumulating a sufficient amount of chitin on the cell surface to strengthen the cell wall.

**Figure 6:**
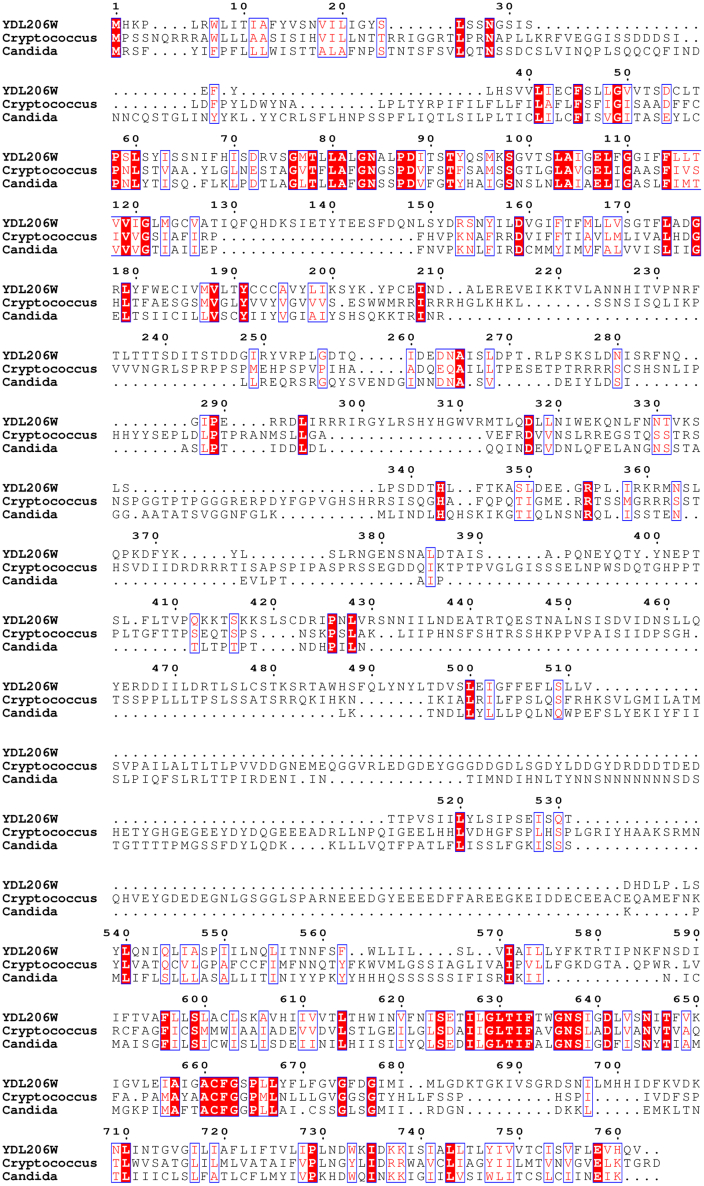
**Alignments of YDL206W and its homologs in pathogenic fungi.** Multiple sequence alignment was conducted for YDL206W and its homologs in *Cryptococcus* (CAALFM_C113490CA) and *Candida* (CaO19.4981). Identical residues are highlighted in *white font* and *red background*, and similar residues are highlighted in *red font*.

Calcium–calcineurin signaling plays a critical role in survival, virulence, and infections of eukaryotic microbial pathogens (8). For instance, it controls the ability of fungal pathogens to grow at host temperature, enables invasive hyphal growth, and strengthens cell wall integrity (8). There are substantial efforts in seeking a better understanding of the regulation of calcium–calcineurin signaling in fungal cells, which could help develop new and safe antifungal drugs by targeting the pathway (40). Our findings here showed that YDL206W is a new component in calcium–calcineurin signaling in yeast, and disrupting the YDL206W gene renders cells unable to grow well at 37 °C. Interestingly, through BLASTp analysis, we find the presence of YDL206W homologs in both *Candida albicans* and *Cryptococcus neoformans*, two important human fungal pathogens (Fig. 6). The top hit from *C. albicans* is CAALFM_C113490CA, whereas the top hit from *C. neoformans* is CNA07800. Notably, both proteins are still uncharacterized. As survival to body temperature of 37 °C is one important virulence factor for human fungal pathogens, it would be very interesting to examine if disrupting the uncharacterized YDL206W homolog in *Candida* and *Cryptococcus* would lead to a similar thermal-sensitive phenotype. If so, they would represent potential targets for developing antifungal drugs for combating these human pathogens.

**Figure 7:**
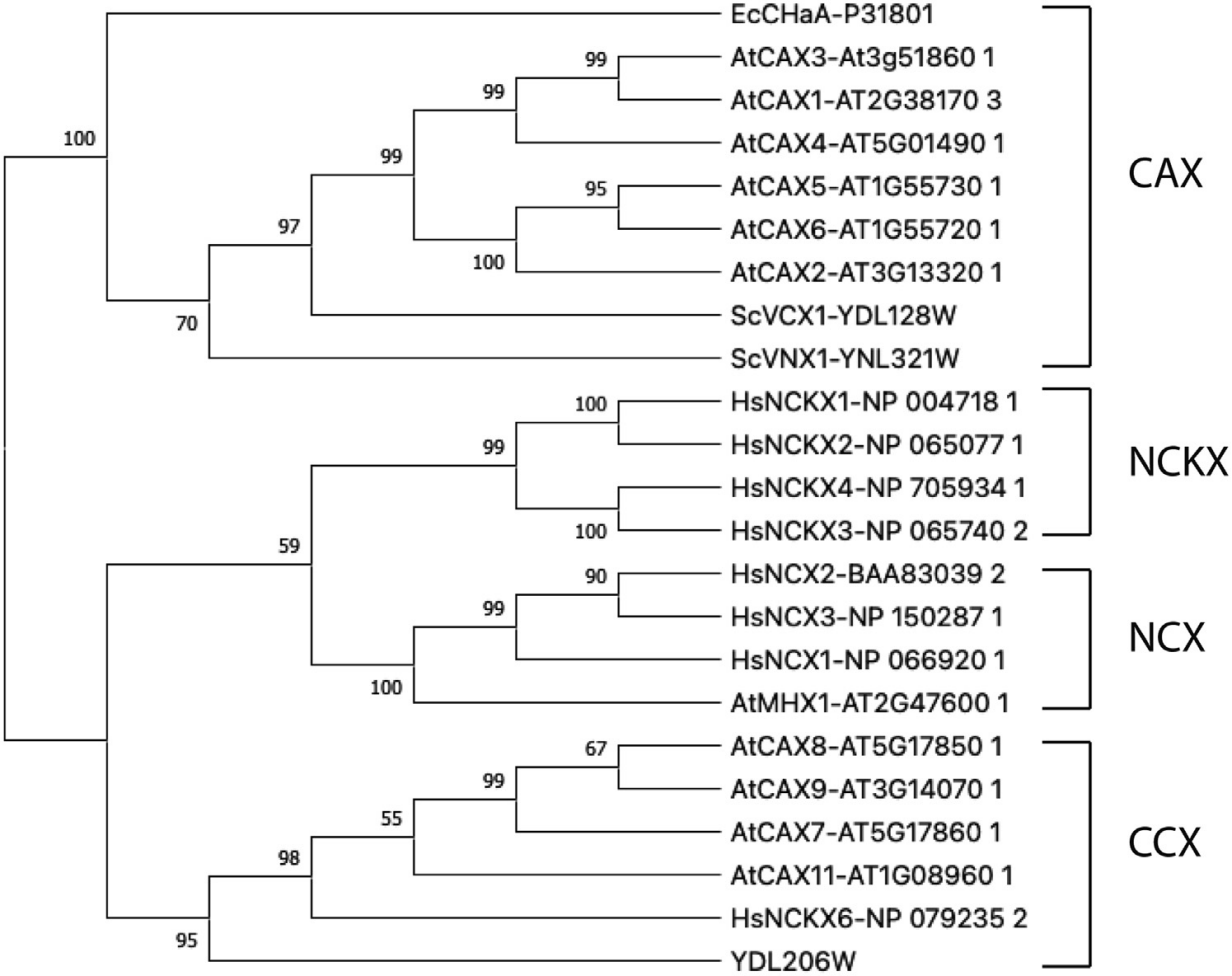
**Phylogenetic analysis.** Protein sequences were acquired according to their accession ID. The phylogenetic tree was inferred using FastTree2 with the maximum likelihood method. The bootstrap values are indicated at the nodes of the branches. At, *Arabidopsis thaliana*; Ec, *Escherichia coli*; Hs, *Homo sapiens*; Sc, *Saccharomyces cerevisiae.*

How does YDL206W contribute to the homeostasis of calcium and calcineurin signaling? YDL206W is predicted to have two NCX domains that are connected with a large loop, a feature shared by many calcium exchangers (24, 41, 42). Phylogenetic studies have defined the existence of four major families within the calcium exchanger superfamily, that is, CAX, NCX, NCKX, and cation/Ca^2+^ exchanger (CCX) (43), in eukaryotes. While members of the NCX family are NCEs, members in the CAX and NCKX families are H^+^/Ca^2+^ exchangers and Na^+^/Ca^2+^–K^+^ exchangers, respectively. CCX is a name given to NCKX6, a calcium exchanger that was initially classified as in the NCKX family (42, 44). To better understand how YDL206W works, we also conducted a phylogenetic analysis of the protein sequence of YDL206W with the sequences of other members of the NCX domain–containing superfamily. Our analysis indicates that YDL206W belongs to the CCX family (Fig. 7). A feature of the CCX family members is the presence of a signature motif in the α-repeat regions that is different from members of the other families (43). As expected, the unique CCX motifs, that is, GNG(A/S)PD in α-1 and (G/S)(N/D)SxGD in α-2, are present in YDL206W (residues 83–89 and residues 636–641) as well as its homologs in *Candida* and *Cryptococcus* (Fig. 6). Thus, it is likely that YDL206W is a cation/Ca^2+^ exchanger in yeast and contributes to the control of intracellular calcium levels. We have thus renamed it Ycx1 for CCX in yeast.

In humans, only one CCX, that is, NCKX6 (also called NCLX), has been identified, whereas in invertebrates, 11 have been identified (41). An initial study suggests that NCKX6 is present in both plasma membrane and ER (42), but later research indicated that NCKX6 is present in mitochondria and plays an important role in mitochondrial calcium efflux (45). Our subcellular localization did not find apparent colocalization of GFP-tagged YDL206W and MitoTracker Red, suggesting YDL206W is not present in mitochondria. Instead, it shows a substantial colocalization with both Sec13-RFP and Anp1-RFP, suggesting its likely localization in ER and Golgi. In yeast, the calcium concentrations in ER and Golgi are around 10 and 200 μM, respectively (46), much higher than that in cytosol. We envision a scenario where in response to the exogenous calcium or pheromone stimulation, YDL206W mediates an increased ER and/or Golgi to cytosol release of Ca^2+^, which contributes to an optimal rise in cytosolic calcium and calcineurin activity. How would these stimuli increase the calcium release from ER and/or Golgi to cytosol? For exogenous calcium, it is possible that the influx of calcium from extracellular space into cytosol may enhance the membrane potential of these organelles, which facilitates the release of calcium into cytosol. This is somewhat analogous to cardiac cells, in which calcium stored in ER is released by trigger calcium, that is, the calcium entered the cells from extracellular space. In mammals, mitochondrial NCXs often form a complex with other proteins such as A-kinase anchoring protein 121 (47, 48) and excitatory amino acid transporter (49), and these NCX-interacting proteins can play an important role in regulating the exchanging activity of mitochondrial NCXs. Thus, alternatively, there may exist YDL206W-interacting proteins in ER and/or Golgi that are important regulators of calcium release.

In summary, our study indicates a clear role of YDL206W, a previously uncharacterized protein with two NCX domains, in maintaining the homeostasis of calcium and regulating signaling by calcineurin. Our work also suggests the possibility of ER and/or Golgi in regulating calcium signaling in yeast. Given the importance of calcium exchangers in health and diseases, future studies on how YDL206W is retained in ER and Golgi and how its activity is regulated would provide insights into the regulation of calcium exchangers in general.

## Experimental procedures

### Strains and plasmids

Standard methods for the growth, maintenance, and transformation of yeast and bacteria and for the manipulation of DNA were used throughout. The yeast *S. cerevisiae* strains used in this study are BY4741 (*MAT****a*** *leu2Δ met15Δ his3Δ ura3Δ*) and BY4741-derived mutants lacking *YDL206W* (Research Genetics), BY4742 (*MAT*α *his3Δ1 leu2Δ0 lys2Δ0 ura3Δ0*)-derived strains expressing RFP-tagged Anp1, Sec13, or Erg6.

The expression plasmids used in this study that have been described previously are *pAMS366-4xCDRE-lacZ* (generously provided by Dr Sabrina Büttner, Stockholm University) (26) and *pVTU-AEQ* (27). The plasmid *pRS313-YDL206W* that expresses YDL206W gene under its own promoter was constructed by a two-step process. The first half containing the promoter and the 5′-end portion of the ORF was amplified and cloned into pRS313 *via* XhoI and ClaI sites. The primers used are 5′-CCG CTC GAG TAA CGA GAT TAC AGT TAC CTC-3′ and 5′-ATC GTC TGT GGA TGT AAT GTC AC-3′. The second half containing the terminator and the 3′-end portion of the ORF was amplified and inserted to the aforementioned plasmid *via* ClaI and BamHI sites. The primers used are 5′-ATC CCT GCG AAA TCA ATG ATG-3′ and 5′-CGC GGA TCC TTA GAC AGA ATC AAA TGA ACA C-3′.

The plasmid *pYES-YDL206W-GFP* that expresses C-terminal GFP-tagged YDL206W under the control of a *GAL1* promoter was constructed by two steps. In the first step, a fragment containing sites for restriction enzymes BamHI, SacI, XhoI, and SalI and followed by the GFP coding sequence was amplified using the primers 5′-GGA TCC GAG CTC GAG GTC GAC ATG AGT AAA GGA GAA GAA C-3′ and 5′-CTA CTA TTT GTA TAG TTC ATC CAT GCC ATG-3′. The amplified fragment was TOPO-cloned into pYES2.1, resulting in pYES2.1-MCS-GFP. The fragment containing YDL206W coding sequences flanked with BamHI and XhoI sites was amplified using the primers 5′-CGC GGA TCC AGA ATG CAT AAA CCT TTA AGA TGG C-3′ and 5′-CCG CTC GAG CAC TTG GTG AAC TTC TAA-3′ and cloned into the pYES2.1-MCS-GFP, resulting in *pYES-YDL206W-GFP*. The plasmid expressing N-terminally GFP-tagged YDL206W was similarly constructed. The GFP coding sequence followed by multiple cloning sites was amplified using the primers 5′-CCA GAA TGA GTA AAG GAG AAG AAC-3′ and 5′-GGA TCC GAG CTC GAG GTC GAC GTC GTG GTC CTT GTA GTC AC-3′ and TOPO-cloned into pYES2.1, resulting in pYES2.1-GFP-MCS. The fragment containing the YDL206W coding sequence flanked with XhoI and BamHI sites was amplified using the primers 5′-CCG CTC GAG ATG CAT AAA CCT TTA AGA TG-3′ and 5′-CGC GGA TCC TTA CAC TTG GTG AAC TTC TA-3′.

### Measurement of cytosolic [Ca^++^]

The level of cytosolic Ca^++^ was measured as described previously (27). Briefly, cells transformed with the plasmid *pVTU-AEQ* expressing aequorin were grown into midlog phase, concentrated 20-fold, and equalized to the same density. The cell numbers were also counted to ensure that the same number of cells were used. Cells were then incubated with 0.02 mg/ml (final concentration) of coelenterazine (from Biotium, Inc) in the dark for 20 min. After three washes to get rid of excess coelenterazine, 100 μl of cell culture was used for the measurement of bioluminescence using the Synergy H1 plate reader. Calcium stimulation was achieved by adding 100 mM (final concentration) of CaCl_2_. Bioluminescence was measured every 14 s, the highest frequency possible by the Synergy H1 plate reader. To measure pheromone-induced calcium rise, we followed the procedure developed previously (28). The level of aequorin expression was examined using Western blotting.

### Western blotting

For all the immunoblotting analyses, midlog phase cells were grown on appropriate medium and then treated or not treated with 80 mM CaCl_2_ or 3 μM alpha factor or 0.5 M NaCl as indicated. Proteins were extracted *via* trichloroacetic precipitation, following procedures described previously (50). Whole cell extracts were resuspended in SDS-PAGE sample buffer (62.5 mM Tris–HCl, pH 6.8, 10% glycerol, 2% SDS, 1% 2-mercaptoethanol, and 0.0005% bromphenol blue) and boiled for 5 min. Following SDS-polyacrylamide gel electrophoresis and transfer to nitrocellulose, the membrane was probed with antibodies to β-galactosidase at 1:1000 dilution (from Promega; catalog no.: Z3783), GFP at 1:5000 dilution (Abcam; catalog no.: ab13970), or aequorin (Abcam; catalog no.: ab9096). Immunoreactive species were visualized by enhanced chemiluminescence detection (Pierce) of horseradish peroxidase–conjugated anti-rabbit immunoglobulin G (Bio-Rad), antirat immunoglobulin G (Abcam), or antichicken immunoglobulin Y (Abcam). All experiments have been repeated at least three times. All experiments were repeated at least three times. Immunoblotting signals were quantified with ImageJ software (National Institutes of Health; https://imagej.nih.gov/ij/download.html). The dot bar graphs were generated using Interactive Dotplot (https://pubmed.ncbi.nlm.nih.gov/28974579/) (51), and the bars represent standard deviations. Where indicated, the data were statistically analyzed by *t* test, with *p* < 0.050 considered significant.

### Microscopy analysis

Cells expressing GFP-tagged YDL206W were grown to midlog phase. Cells were concentrated, and 10 μl of concentrated cell suspension was placed on a slide with a thin layer of 0.5% agar and visualized by fluorescence microscopy using an Olympus FV1000 laser-scanning confocal microscope. For Calcofluor white staining, 10 ml of midlog phase cells grown in SCD media was concentrated to 200 μl. After addition of 2 μl of Calcofluor white solution (1 mg/ml; Sigma–Aldrich; catalog no.: 18909) and 2 μl of 1 M KOH, the cells were imaged using a confocal microscope. The same exposure settings were used for capturing the images to ensure consistency.

### Phylogenetic analysis

Protein sequences were acquired from the National Center for Biotechnology Information, *Saccharomyces* Genome Database, and the Arabidopsis Information Resource according to their accession ID. Multiple sequence alignments were performed by MAFFT (https://pubmed.ncbi.nlm.nih.gov/28968734/), version 7 online service (52, 53). The phylogenetic tree was inferred using the FastTree2 (https://journals.plos.org/plosone/article?id=10.1371/journal.pone.0009490) with the maximum likelihood method, and the bootstrap value was indicated at the node of the branches (54).

## Data availability

All data are available in the main text or the supporting information.

## Supporting information

This article contains supporting information (Fig. S1).

## Conflict of interest

The authors declare that they have no conflicts of interest with the contents of this article.
