# Supplementary material for: The Ycx1 protein encoded by the yeast YDL206W gene plays a role in calcium and calcineurin signaling
Source: J Biol Chem. 2023 Mar 24;299(5):104647. doi: 10.1016/j.jbc.2023.104647 (PMC10126930; doi:10.1016/j.jbc.2023.104647)
Supplement: Supplemental Figure S1 [file mmc1.pdf]

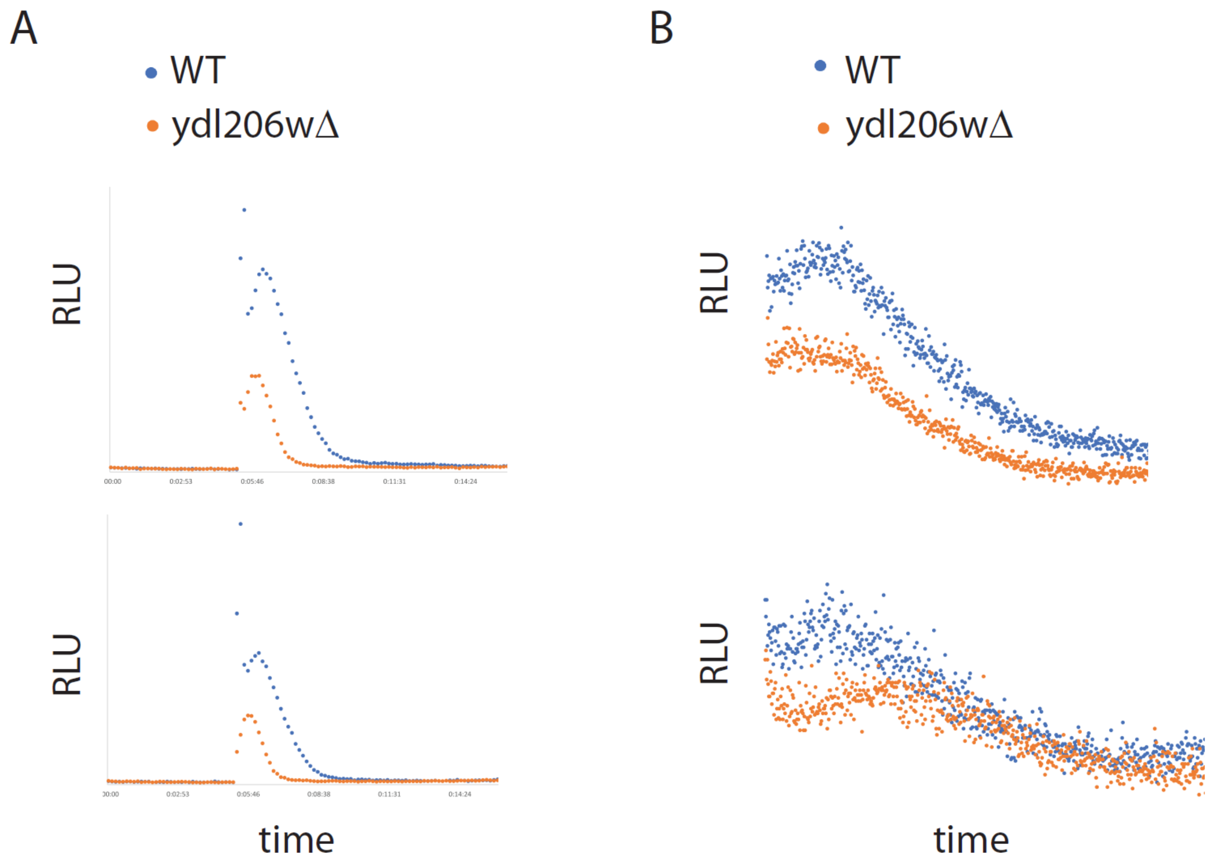

Figure S1. Intracellular calcium level. *A*, wild type or *ydl206wΔ* cells transformed with a plasmid expressing apo-aequorin were grown to mid-log phase. Equal amounts of cells were incubated with coelenterazine for 30 minutes. After a stable baseline measurement of bioluminescence was achieved, cells were treated with 80 mM $CaCl_2$ and the resulting bioluminescence was recorded every 14 seconds. *B*, the same cells as described in panel *A* were grown to mid-log phase, treated with 3 µM α-factor for 15 min and incubated with coelenterazine for 30 min, and the bioluminescence was measured every 14 seconds. Results from two independent experiments are shown.
